# *U2AF2* Missense Variant Associated With Epilepsy and Systemic Dysmorphism: Report of Two Cases and Literature Review

**DOI:** 10.1002/ccr3.71182

**Published:** 2025-10-12

**Authors:** Shiqin Huang, Mei Li, Hai Yuan, Yunli Han, Xiaolan Chen, Yunxuan Su, Xing Li

**Affiliations:** ^1^ Departments of Pediatrics The First Affiliated Hospital of Guangxi Medical University Nanning China; ^2^ Departments of Pediatrics Nanning Maternal and Child Health Care Hospital Nanning China

**Keywords:** epilepsy, missense variant, neurodevelopment, systemic dysmorphism, *U2AF2*

## Abstract

Recurrent *U2AF2* c.445C>T variant in two cases with systemic dysmorphism, epilepsy, and neurodevelopmental regression suggests a novel spliceosomal gene‐brain disorder link, warranting *U2AF2* screening in unexplained neurodevelopmental cases.

## 1 Introduction

U2 auxiliary factor (U2AF) is a non‐snRNP protein that is crucial for the binding of U2 snRNP to the pre‐mRNA branch site. It serves as a fundamental pre‐mRNA splicing factor in an early stage of splicing [1, 2]. Mutations or dysregulation of numerous genes leading to aberrant splicing can result in neurodevelopmental disorders [3, 4, 5]. *U2AF2* is a vital pre‐mRNA splicing factor in the initial phase of splicing [1]. Missense variants in this gene have been reported to cause systemic dysmorphism, developmental delay, and epilepsy [6, 7, 8, 9, 10]. Specifically, a large trio whole‐exome sequencing (WES) study involving patients with developmental disorders identified *U2AF2* as a potential pathogenic gene. Ten patients with seven variant types, including p.(Arg149Trp), p.(Arg149Gln), p.(Arg150Cys), p.(Val153Met), p.(Arg150His), p.(Val186Met), and p.(Gly265Asp), were identified, although detailed clinical information was not provided [6, 11]. Currently, several case reports have described patients with developmental disorders where *U2AF2* was identified as a pathogenic gene. The most common pathogenic variants reported were c.603G>T p.(E163_E201del) and c.445C>T p.(Arg149Trp) [7, 8, 9, 10]. Recently, *U2AF2* and two other splicing factors were found to establish a genetic network underlying human brain development and function [11], confirming the indispensable role of this gene in neural development. Herein, we report on two Chinese patients harboring the same variant in *U2AF2*, who exhibit typical features of global developmental delay, dysmorphic facial features, and epilepsy. The detailed clinical manifestations of these two cases provide further evidence of the crucial role played by *U2AF2* in neural development.

## 2 Case Examination

**FIGURE 1:**
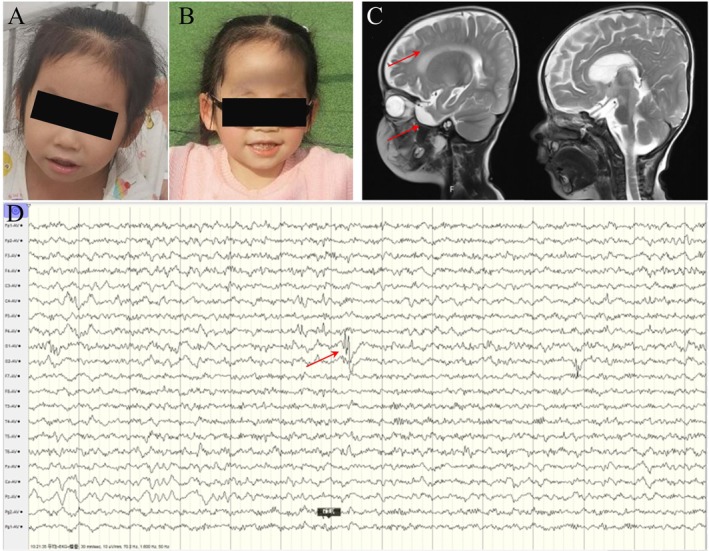
(A, B) Clinical photographs of the patient. (C) Sagittal MR images of the head; red arrows denote abnormal anatomical structures. (D) EEG tracing; the red arrow indicates an episode of abnormal electrical discharge.

Case 1 involved an 8‐year‐old girl of the Zhuang ethnic group. She was born to non‐consanguineous parents after 39 weeks of gestation without asphyxia, weighing 2690 g, with a body length of 48 cm and a head circumference of 32 cm. She has an older sibling who is unaffected. Her parents are healthy and have no family history of neurodevelopmental disorders. Following her birth, due to delayed motor development compared to her peers, she underwent continuous rehabilitation treatment and repeatedly experienced respiratory infections. At the age of 1, her vision was tested and found to be hyperopic. When she was 3 years and 10 months old, she experienced her first epileptic seizure without fever, presenting as a persistent state of generalized tonic and clonic activity lasting up to 30 min. Abnormal physical features included microcephaly and global developmental delay. Dysmorphic facial features included large ears, frontal bossing, wide eye spacing, bilateral ptosis, wide and sparse eyebrows, and underdeveloped teeth (Figure 1A,B). The patient has experienced recurrent seizures for over 3 years, resulting in focal seizures and a persistent state of multiple seizures. She started valproic acid treatment at the age of 3. At the age of 6 years and 11 months, she was admitted to the hospital due to recurrent convulsions accompanied by a fever of 40°C. Brain magnetic resonance imaging (MRI) revealed abnormalities characterized by small frontal and temporal gyri on both sides, corresponding widening of the subdural space, and a small size of the corpus callosum (Figure 1C). The electroencephalogram (EEG) was abnormal, showing slow background activity. During wakefulness, bilateral anterior slow wave emissions were observed, more significant on the left side, and during sleep, bilateral central and parietal spike‐slow wave emissions were noted (Figure 1D). The patient presented with generalized tonic–clonic seizures (GTCS), characterized by double‐eyed gaze, cyanosis of the lips, closed teeth, stiffness and trembling of the limbs, inability to respond, and self‐relief after about 3–4 min. Visual evoked potential testing showed prolonged latency of P100 waves in the full field, indicating central damage. The Wechsler Children's Intelligence scale indicated that she had an intellectual disability and moderate social maladaptation.

**FIGURE 2:**
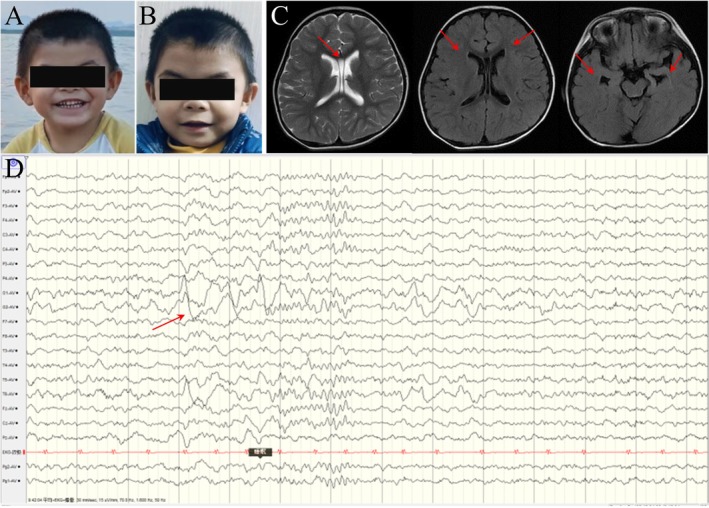
(A, B) Clinical photographs of the patient. (C) Axial MR images of the brain; red arrows denote regions with abnormal signals or structural changes. (D) EEG tracing; the red arrow indicates episodes of abnormal electroencephalographic.

Case 2 presented was a 6‐year‐old boy of the Zhuang ethnic group. He was delivered via cesarean section at 36 weeks gestation due to fetal bradycardia, with no history of resuscitation for asphyxia. At birth, his weight was 2000 g, length was 44 cm, and head circumference was 30 cm. Postnatal echocardiographic evaluation revealed cardiac murmurs. At 3 months of age, further echocardiographic assessment demonstrated a 5 mm ventricular septal defect (VSD) and multiple atrial septal defects (ASDs), totaling three defects with sizes of 5 × 7, 5 × 5, and 3 × 3 mm, respectively. Follow‐up echocardiograms at 1 and 3 years of age confirmed the presence of multiple secundum ASDs and spontaneous closure of the VSD. The patient's motor development lagged behind his peers by approximately 2–3 months. Postnatally, neutropenia was noted, prompting treatment with vitamin B4. Subsequent neutrophil counts ranged from 0.79 to 1.35 × 10^9/L, accompanied by recurrent respiratory infections and multiple episodes of pneumonia. The patient exhibited a distinct facial phenotype similar to a previously described female patient, characterized by a protruding forehead, wide eye spacing, large ears, and broad, sparse eyebrows (Figure 2A,B). At 1 year and 6 months of age, the patient experienced his first epileptic seizure, manifesting as a persistent focal seizure state with loss of consciousness, upward deviation of the eyes, staring to the right, and occasional right‐sided oral and limb tremors. These seizures were not associated with fever and lasted for approximately 1 h. Symptoms were alleviated with Midazolam infusion. At 2 years of age, the patient experienced another seizure and was admitted to the hospital for oral administration of levetiracetam. By 4 years and 6 months of age, the patient's physical development was below average, with a weight of 13 kg (< 3 standard deviations (SD) below the mean) and a height of 95 cm (< 3 SD below the mean). Development scale assessments yielded scores of 46 for speech, 57 for fine motor skills, 47 for the total scale, and 8 for social adaptability. Head MRI revealed an enlarged septum pellucidum cavity (6 mm), inflammation in the right maxillary and ethmoid sinuses, and adenoid hypertrophy (Figure 2C). EEG analysis showed increased background theta activity, interspersed with spikes/sharp waves, sharp slow waves, and irregular slow waves emanating from the right posterior head during sleep (Figure 2D). Routine blood tests and biochemical analyses were unremarkable, with normal blood gas, lactate, blood sugar, and electrolyte levels.

## 3 Methods

Electroencephalography monitoring was conducted using a 64‐channel electroencephalograph manufactured by EEG‐1200C (Nihon Kohden Corporation, Japan). Cranial MRI was performed using a 1.5 T MR System (Siemens MAGENTOM Altea, Germany). Genomic DNA was isolated from peripheral blood samples of the proband and their parents. Trio WES was performed using the NextSeq500 platform (Illumina, USA). The mean depth of coverage across the entire exome exceeded 100×, with over 99% coverage of the target area. Sanger sequencing was used to validate the variants. The pathogenicity of the variants was predicted using PolyPhen‐2, SIFT, CADD, and Mutation Taster. Variants were evaluated in the context of populations represented in the 1000 Genomes Project, ExAC, and gnomAD databases. The pathogenicity of each variant was assessed according to the guidelines established by the American College of Medical Genetics and Genomics (ACMG).

## 4 Conclusion and Results

**FIGURE 3:**
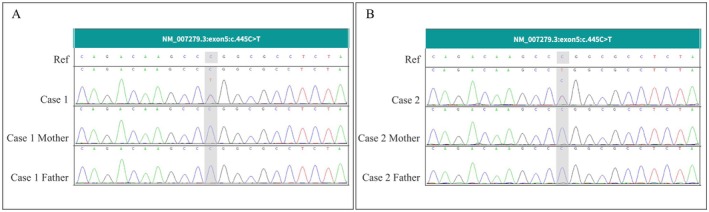
Sanger sequencing map. (A) The case 1 Sanger sequencing map; (B) The case 2 Sanger sequencing map. Ref: Reference sequence. Case 1 Case 2: Sequencing results of the proband in each case. Case 1 Mother/Case 2 Mother: Sequencing results of the proband's mother in each case. Case 1 Father/Case 2 Father: Sequencing results of the proband's father in each case. Sanger sequencing results for the NM_007279.3:Exon5:C.445C>T variant in two cases (A, B). Panels show sequencing traces of the reference sequence (Ref), probands (Case 1, Case 2), and their respective parents (Case 1 Mother, Case 1 Father; Case 2 Mother, Case 2 Father). The gray shaded area highlights the position of the c.445C>T variant. The reference genome used is the human reference genome GRCh38/hg38.

During the in‐depth investigation of diseases and their pathogenic mechanisms, we conducted trio WES on two familial cases. The sequencing results revealed that both cases harbored a c.445C>T p.(Arg149Trp) mutation in the *U2AF2* gene (NM_007279.3), which is classified as a heterozygous missense mutation. Multiple protein function damage prediction software tools were utilized for assessment, all consistently predicting this mutation to have a “deleterious” effect. Further Sanger sequencing validation confirmed that both parents of the probands were wild‐type at this locus (Figure 3A,B), indicating that this mutation is a de novo mutation in both cases. By searching normal population control databases, including gnomAD, ExAC, and 1000Genomes, no frequency reports of this variant were found, further supporting its rarity. Following the guidelines and standards of the ACMG, we conducted a detailed rating evidence analysis for this variant site “PM5, PM2‐Supporting, PM1, PS4‐Strong, PS2‐Moderate, PP2” and classified and rated it accordingly “Pathogenic.” The noteworthy point is that the U2AF2 gene is linked to a specific disease phenotype in the Online Mendelian Inheritance in Man (OMIM) database: developmental delay, dysmorphic facies, and brain anomalies (MIM: 620535). The mode of inheritance is autosomal dominant (AD), which closely corresponds to the phenotypic manifestations observed in the patients of both cases.

## 5 Discussion

Disordered RNA splicing can lead to the occurrence of various human diseases, including spinocerebellar ataxias, spinal muscular atrophy, myotonic dystrophy, retinitis pigmentosa, and autoimmune disease [12, 13]. The U2 auxiliary factor (U2AF) is a non‐snRNP protein essential for the binding of U2 snRNP to the pre‐mRNA branch site, and it can guide the early stages of splice‐site choice by recognizing polypyrimidine tract consensus sequences adjacent to the 3′‐splice site [1, 2]. Intron removal from pre‐mRNA through splicing is crucial for RNA metabolism, generating proteomic diversity from a limited gene set [14]. Recently, de novo mutations in *U2AF2* have been implicated in neurodevelopmental disorders [6, 7, 8, 10].

A large trio WES study focusing on patients with developmental disorders identified *U2AF2* as a potential pathogenic gene, with ten patients harboring seven variant types, albeit without detailed follow‐up clinical information [6]. Several case reports have now described *U2AF2* as a pathogenic gene in patients with developmental disorders. Notably, the *U2AF2* variant c.445C>T p.(R149W) has been reported in two cases of neurodevelopmental disorders [7, 8, 10]. The patient involved was a 7.5‐year‐old Japanese girl with developmental delay, dysmorphic features, and brain anomalies [7]. She carried a de novo heterozygous mutation c.445C>T in the *U2AF2* gene, resulting in an Arg149‐to‐Trp (R149W) substitution in a conserved residue within RNA recognition motif 1 (RRM1). This patient exhibited global developmental delay, intellectual disability, epilepsy, short stature, microcephaly, facial dysmorphism, intermittent exotropia, bilateral ptosis, muscle hypotonia, and a thin corpus callosum, suggesting that *U2AF2*‐related disorders may encompass systemic dysmorphisms, epilepsy, and brain malformations, along with global developmental delay. The mutation, identified by trio‐based WES and confirmed by Sanger sequencing, was absent from the gnomAD database. Subsequently, the same variant in *U2AF2* was identified in a 4‐year‐old Caucasian male [8]. Presenting similar symptoms of global developmental delay, seizures, and short stature. These findings strongly support that *U2AF2* is a pathogenic gene and that the c.445C>T variant can cause a distinct neurodevelopmental disorder. Very recently, additional reports have further solidified the association between *U2AF2* and neurodevelopment.

A 6‐year‐old Chinese boy harboring a de novo heterozygous c.603G>T (NM_007279.3) transversion in the last nucleotide of exon 6 of the *U2AF2* gene displayed a clinical phenotype similar to previously reported patients, including epilepsy, intellectual disability, language delay, microcephaly, and hypoplastic corpus callosum [10]. RT‐PCR analysis of patient cells revealed abnormal splicing and skipping of exon 6, resulting in a 39‐residue in‐frame deletion (E163_E201del). These cells exhibited a 50% reduction in wild‐type *U2AF2* mRNA and protein (65‐kD) expression compared to controls, with a smaller 55‐kD band detected, corresponding to the abnormal protein with the in‐frame deletion. The mutation significantly inhibited the proliferation of patient‐immortalized lymphocytes, shifting the cell cycle distribution with an increased proportion of cells in the G1/G0 phase and a decreased proportion in the G2/M phase compared to controls. Moreover, a de novo heterozygous variant in *U2AF2* c.470C>T p.(Pro157Leu) was identified by trio exome sequencing in a 2‐year‐old Japanese girl diagnosed with hypomyelinating leukodystrophy and global developmental delay [9].

In summary, two patients with the *U2AF2* gene variant causing systemic malformations and epilepsy have been reported, both presenting with status epilepticus, epilepsy, global developmental delay, and significant abnormalities in electroencephalography and MRI. Second‐generation sequencing confirmed the presence of a heterozygous variant in *U2AF2* c.445C>T p.(Arg149Trp) in both cases. Our findings suggest that *U2AF2*‐related disorder may encompass systemic dysmorphisms, epilepsy, and brain malformations, along with developmental delay and intellectual disability, providing robust evidence that *U2AF2* is a pathogenic gene and that the c.445C>T p.(Arg149Trp) variant can lead to a unique neurodevelopmental disorder.

## Author Contributions

**Shiqin Huang:** data curation, writing – original draft, writing – review and editing. **Mei Li:** formal analysis, visualization, writing – original draft. **Hai Yuan:** software, validation, visualization. **Yunli Han:** software, validation, visualization. **Xiaolan Chen:** conceptualization, writing – review and editing. **Yunxuan Su:** investigation. **Xing Li:** conceptualization, funding acquisition, project administration, supervision, writing – original draft, writing – review and editing.

## Conflicts of Interest

The authors declare no conflicts of interest.

## Data Availability

The data that support the findings of this study are available on request from the corresponding author. The data are not publicly available due to privacy or ethical restrictions.
